# Pharmacological SIRT2 Inhibition by AGK2 Attenuates Hapten-Induced Keratinocyte Inflammation via NF-κB/NLRP3 Suppression and Nrf2 Reactivation

**DOI:** 10.5812/ijpr-169951

**Published:** 2026-05-04

**Authors:** Xianhua Qiao, Xinglong Yu, Juanjuan Gao, Chong Lv, Fang Cheng

**Affiliations:** 1 Department of Dermatology，Xingtai People's Hospital, Xingtai, China; 2 Department of Information Engineering，Hebei Vocational University of Technology and Engineering, Xingtai，China

**Keywords:** SIRT2, AGK2, pharmacological inhibition, keratinocytes, allergic contact dermatitis, NF-κB, NLRP3 inflammasome, Nrf2, oxidative stress, host-directed therapy.

## Abstract

**Background:**

Allergic contact dermatitis (ACD) is initiated by hapten-induced keratinocyte oxidative stress and inflammatory signaling, commonly involving NF-κB activation, NLRP3 inflammasome priming–associated signaling, and inadequate Nrf2-dependent antioxidant responses. Identification of druggable upstream regulators that integrate these pathways could support host-directed pharmacological strategies for dermatitis-like inflammation.

**Objective:**

To investigate whether pharmacological inhibition of SIRT2 using AGK2 mitigates hapten-triggered inflammatory–oxidative stress signaling in human keratinocytes and to define the associated molecular mechanisms.

**Methods:**

Human Ker-CT keratinocytes were cultured alone or in transwell co-culture with CCD-1064Sk dermal fibroblasts and stimulated with 2,4-dinitrochlorobenzene (DNCB); nickel sulfate served as a comparator sensitizer. Cells were treated with the selective SIRT2 inhibitor AGK2, and target engagement was verified by acetyl-α-tubulin accumulation. Outcomes included viability (MTT), ROS generation (DCFDA), cytokine expression/secretion (qPCR/ELISA), and pathway signaling (Western blotting, immunofluorescence, nuclear–cytoplasmic fractionation), focusing on NF-κB (p65/IκBα), NLRP3 priming–associated readouts (NLRP3 expression and IL-1 family cytokine output), and Nrf2 localization with downstream antioxidant enzymes (HO-1, SOD). To strengthen specificity beyond pharmacological inhibition, SIRT2 involvement was genetically validated using siRNA-mediated knockdown (siSIRT2) in Ker-CT cells. In addition, AGK2-alone control groups (without DNCB) were included across major readouts to assess baseline effects on NF-κB, ROS, and Nrf2 signaling, and Keap1 expression was examined as mechanistic support for altered Nrf2 regulation.

**Results:**

DNCB produced a reproducible sub-toxic keratinocyte stress phenotype characterized by increased ROS, activation of NF-κB signaling with IκBα degradation and p65 nuclear translocation, upregulation of NLRP3 expression with elevated IL-1β/IL-18 secretion consistent with enhanced inflammasome-associated inflammatory responses, and impaired Nrf2 nuclear accumulation with reduced antioxidant enzyme expression. AGK2-mediated SIRT2 inhibition significantly attenuated NF-κB activation and reduced pro-inflammatory cytokine outputs, while reducing NLRP3 priming–associated responses and lowering IL-1β/IL-18 secretion. In parallel, AGK2 restored Nrf2 nuclear translocation and enhanced expression of HO-1 and SOD, resulting in partial normalization of intracellular redox balance. Importantly, siSIRT2 phenocopied AGK2 effects by reducing DNCB-induced NF-κB activation, ROS accumulation, and restoring nuclear Nrf2 enrichment. AGK2 alone did not significantly alter basal NF-κB signaling, ROS levels, or Nrf2 nuclear localization, supporting that AGK2 effects were stimulus-dependent rather than intrinsic pathway suppression. Consistent with impaired antioxidant competence under DNCB challenge, Keap1 levels increased with DNCB and were partially normalized by SIRT2 inhibition/knockdown, providing mechanistic context for the ROS–Nrf2 paradox.

**Conclusion:**

Pharmacological inhibition of SIRT2 with AGK2 is associated with anti-inflammatory and antioxidant effects in hapten-stimulated human keratinocytes, including attenuation of NF-κB signaling and reduction of NLRP3 priming–associated IL-1 family cytokine responses, together with restoration of Nrf2-dependent cytoprotective signaling. Collectively, these findings suggest that SIRT2 may have pharmacological relevance in ACD-like inflammatory skin stress and warrant further evaluation in preclinical dermatitis models and therapeutic development studies.

## 1. Introduction

Allergic contact dermatitis (ACD) is a common, immune-mediated inflammatory skin disease that arises when low-molecular-weight haptens penetrate the epidermis, bind to self-proteins, and elicit a type IV hypersensitivity reaction upon re-exposure (1 - 3). Clinically, ACD manifests as erythema, edema, scaling, and pruritic eczematous lesions that may become chronic and relapsing, leading to substantial impairment of quality of life and work productivity (4,5).

At the cellular level, ACD is initiated at the keratinocyte–dermal fibroblast interface, where chemical sensitizers provoke oxidative stress, pro-inflammatory cytokine release, and activation of innate immune signaling cascades that shape downstream adaptive T-cell responses (6,7). Despite the extensive use of ACD models in dermatology and toxicology, the early keratinocyte-centered molecular events that integrate redox imbalance, inflammasome engagement, and stress-responsive signaling remain incompletely defined, limiting identification of pharmacologically actionable nodes for host-directed intervention (8,9).

Model haptens such as 2,4-dinitrochlorobenzene (DNCB) and metal allergens such as nickel are widely used to reproduce key features of ACD in vitro and in vivo because they reliably induce keratinocyte activation, cytokine release, and oxidative stress (10 - 12). In keratinocytes, DNCB rapidly elevates reactive oxygen species (ROS), activates NF-κB and mitogen-activated protein kinase signaling, and promotes secretion of TNF-α, IL-1β, and related pro-inflammatory mediators (13 - 15). In parallel, engagement of pattern-recognition signaling and ion flux–dependent events can promote NLRP3 inflammasome priming and inflammasome-associated inflammatory responses, reflected by increased NLRP3 expression and enhanced IL-1 family cytokine output; however, definitive confirmation of full inflammasome activation requires additional complex-level readouts such as caspase-1 and/or gasdermin D cleavage (16,17).

Keratinocytes also deploy endogenous antioxidant defense mechanisms, most prominently the Nrf2–HO-1–SOD axis, which limits oxidative damage by inducing cytoprotective and detoxifying enzymes (18). The functional balance between NF-κB and NLRP3 priming–associated inflammatory amplification and Nrf2-mediated cytoprotection is therefore a key determinant of whether hapten exposure resolves as transient irritation or progresses toward sustained pathological inflammation.

Sirtuins are NAD⁺-dependent deacetylases that act as metabolic and stress sensors. Among them, Sirtuin-2 (SIRT2) is predominantly cytosolic but can shuttle to the nucleus, where it regulates diverse substrates including α-tubulin, histones, transcription factors, and inflammasome-associated proteins (19). SIRT2 has been implicated in neuroinflammation, metabolic disorders, and systemic inflammatory responses, where its inhibition attenuates NF-κB signaling, suppresses pro-inflammatory cytokine production, and modulates redox homeostasis (20). However, whether SIRT2 represents a pharmacologically relevant regulator of contact sensitizer–driven keratinocyte inflammation in the cutaneous setting remains largely unexplored.

AGK2 is a relatively selective small-molecule inhibitor of SIRT2 widely used to interrogate SIRT2 function. AGK2 inhibits SIRT2 deacetylase activity—classically evidenced by accumulation of acetylated α-tubulin—and modulates downstream inflammatory and oxidative stress signaling outputs (21). Although SIRT2 inhibition has shown protective effects in inflammatory and oxidative injury contexts, its mechanistic contribution within a DNCB-driven, ACD-like keratinocyte–fibroblast co-culture system has not been systematically examined.

In this study, we established a transwell co-culture model using human Ker-CT keratinocytes and CCD-1064Sk dermal fibroblasts to better recapitulate epidermal–dermal microenvironmental signaling during hapten challenge. Using DNCB as the primary sensitizer and nickel sulfate as a comparator for initial model validation, we investigated whether SIRT2 functions as an upstream regulatory node integrating NF-κB–driven inflammatory signaling, NLRP3-associated IL-1 family cytokine responses, and Nrf2-dependent antioxidant competence under ACD-like conditions. Beyond pharmacological inhibition with AGK2 (validated by acetyl-α-tubulin accumulation), we incorporated siRNA-mediated SIRT2 knockdown to genetically confirm SIRT2 involvement and included AGK2-alone groups (without DNCB) to define baseline effects on NF-κB, ROS, and Nrf2 signaling. By integrating viability assays, ROS measurements, cytokine profiling, Western blotting, immunofluorescence, and nuclear–cytoplasmic fractionation with mechanistic assessment of Keap1–Nrf2 regulation, we delineate a SIRT2-dependent inflammatory–redox circuit in DNCB-stimulated keratinocytes and provide a drug-mechanistic rationale for targeting SIRT2 to rebalance pathogenic inflammation and endogenous cytoprotection in allergic contact dermatitis.

## 2. Materials and Methods

### 2.1. Cell Lines and Culture Conditions

Human keratinocytes (Ker-CT) and human dermal fibroblasts (CCD-1064Sk) were cultured at 37°C in a humidified incubator with 5% CO₂. Cells were maintained in high-glucose Dulbecco’s modified Eagle’s medium (DMEM) supplemented with 10% heat-inactivated fetal bovine serum (FBS), 1% penicillin–streptomycin, and 2 mM L-glutamine. Cells were passaged at 70 - 80% confluence using 0.05% trypsin–EDTA and used between passages 5 and 20. All cultures were routinely screened and confirmed to be mycoplasma-free by PCR-based assays.

### 2.2. Transwell Co-culture System

To model dermal–epidermal interactions relevant to allergic contact dermatitis, Ker-CT keratinocytes were co-cultured with CCD-1064Sk fibroblasts using polyester transwell inserts (0.4 μm pore size). Fibroblasts were seeded in the lower compartment (8.0 × 10⁴ cells/well in 12-well plates), while keratinocytes were seeded in the upper inserts at 8.0 × 10⁴ cells/insert (12-well format). Co-cultures were equilibrated for 24 h prior to treatments. Monoculture controls were processed in parallel under identical conditions (22,23).

### 2.3. Reagents and Experimental Treatments

**Figure 1.**
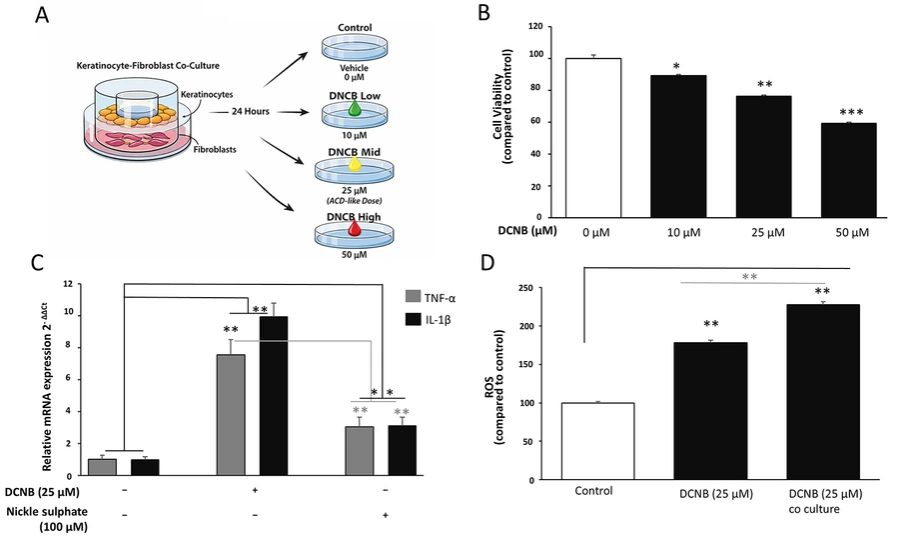
Establishment of an ACD-like inflammatory and oxidative stress model in Ker-CT keratinocytes A, Schematic representation of the in vitro allergic contact dermatitis–like model. Ker-CT keratinocytes were cultured either as monocultures or in transwell co-culture with CCD-1064Sk fibroblasts and exposed to DNCB. Nickel sulfate was included as a comparator sensitizer for model validation; subsequent mechanistic analyses focused on DNCB due to its stronger and more reproducible phenotype. B, Cell viability of Ker-CT keratinocytes following exposure to DNCB (10, 25, 50 μM; 24 h) was assessed by MTT and normalized to vehicle control (set to 100%). C, TNF-α and IL-1β mRNA expression following DNCB or nickel sulfate treatment was quantified by RT-qPCR, normalized to GAPDH, and expressed as fold change relative to vehicle control. Nickel sulfate was used only for model validation and was not extended to downstream pathway analyses. D, Intracellular ROS levels were measured by DCFDA fluorescence in Ker-CT monoculture or Ker-CT/CCD-1064Sk transwell co-culture following DNCB exposure and normalized to vehicle control. Data are mean ± SD from ≥ 3 independent biological experiments (n ≥ 3). Statistical testing: one-way ANOVA with Tukey’s post hoc test. Significance: *P < 0.05, **P < 0.01, ***P < 0.001. Where shown, horizontal comparison bars denote statistical contrasts (black bars: vs vehicle control; grey bars: vs DNCB alone).

2,4-Dinitrochlorobenzene (DNCB), nickel sulfate hexahydrate, AGK2 (selective SIRT2 inhibitor), and BAY 11 - 7082 (NF-κB inhibitor) were obtained from commercial suppliers. DNCB and AGK2 were dissolved in DMSO, with final solvent concentrations maintained at ≤ 0.1% (v/v). Nickel sulfate was dissolved in sterile culture medium. Nickel sulfate was included as a comparator sensitizer exclusively for initial model validation to confirm allergen-responsiveness of the co-culture system (Figure 1). Subsequent mechanistic experiments focused on DNCB because it produced a more robust and reproducible inflammatory/oxidative phenotype in Ker-CT cells, providing a wider dynamic range for interrogation of SIRT2-dependent NF-κB/NLRP3/Nrf2 signaling.

Based on dose–response and cytotoxicity screening, DNCB was used at 25 μM for mechanistic studies. Specifically, 25 μM was selected because it reproducibly induced ROS and inflammatory signaling while remaining sub-toxic in Ker-CT cells in our initial MTT titration (10, 25, 50 μM), thereby providing an appropriate dynamic range for pathway analyses. AGK2 was applied at 10 μM, a concentration widely reported to effectively inhibit SIRT2 without inducing cytotoxicity. In our system, 10 μM AGK2 produced clear target engagement (increased acetyl-α-tubulin) without altering basal NF-κB/ROS/Nrf2 readouts in AGK2-alone controls, supporting its use as a non-cytotoxic, on-target inhibitory dose. BAY 11 - 7082 was used at 5 μM. In inhibitor experiments, cells were pretreated for 1 h before DNCB exposure. Unless otherwise stated, all treatments were performed for 24 h. Accordingly, nickel sulfate was not carried forward into downstream pathway analyses and was used only as an early comparator control.

### 2.4. Cell Viability Assay (MTT)

Ker-CT cells were seeded in 96-well plates (1.0 × 10⁴ cells/well in 100 µL medium) and treated with graded concentrations of DNCB (10, 25, and 50 μM). MTT reagent (5 mg/mL) was added for 3 - 4 h, and formazan crystals were solubilized with DMSO. Absorbance was measured at 570 nm. Viability was normalized to vehicle controls and expressed as percentage of control (24).

### 2.5. siRNA-Mediated SIRT2 Knockdown

For genetic validation experiments Ker-CT keratinocytes were transfected with SIRT2-targeting siRNA (siSIRT2) or non-targeting control siRNA (siCtrl) using a lipid-based transfection reagent according to the manufacturer’s instructions. Transfections were performed in Ker-CT cells only (upper insert) and allowed to proceed for 24 - 48 h to achieve protein knockdown before DNCB stimulation. Following knockdown, transfected keratinocytes were assembled into the transwell co-culture system with CCD-1064Sk fibroblasts and subjected to the treatment conditions described below. Knockdown efficiency and functional target engagement were verified by SIRT2 immunoblotting and acetyl-α-tubulin accumulation.

### 2.6. Measurement of Intracellular ROS

Ker-CT cells were seeded at 1.5 × 10⁴ cells/well in black 96-well plates for DCFDA assays for fluorescence normalization experiments. ROS levels were quantified using DCFDA (10 - 20 μM, 30 min incubation).

### 2.7. RNA Extraction and Quantitative PCR

Ker-CT cells were seeded at 2.5 × 10⁵ cells/well in 6-well plates for RNA isolation. Total RNA was isolated using TRIzol reagent and reverse transcribed using a cDNA synthesis kit. qPCR was performed using SYBR Green chemistry. Target genes included TNF-α, IL-1β, NLRP3, HMOX1 (HO-1), and SOD1, with GAPDH as internal control. Relative expression was calculated using the 2⁻ΔΔCt method.

Primer sequences (5’→3’) for TNF-α, IL-1β, NLRP3, HMOX1, SOD1, and GAPDH are provided in Table 1

**Table 1.** Primer Sequences (5′→3′) Used for RT-qPCR Analysis of Target Genes

Target Gene	Forward Primer (5′→3′)	Reverse Primer (5′→3′)
TNF-α (TNF)	CCTCTCTCTAATCAGCCCTCTG	GAGGACCTGGGAGTAGATGAG
IL-1β (IL1B)	ATGATGGCTTATTACAGTGGCAA	GTCGGAGATTCGTAGCTGGA
NLRP3	GATCTTCGCTGCGATCAACA	GGGATTCGAAACACGTGCATTA
HO-1 (HMOX1)	TATCGTGCTCGCATGAACACT	CCAACACTGCATTTACATGGC
SOD1	AAGGCCGTGTGCGTGCTGAA	CAGGTCTCCAACATGCCTCT
GAPDH	GAAGGTGAAGGTCGGAGTC	GAAGATGGTGATGGGATTTC

Table 1. Primer sequences (5′→3′) used for RT-qPCR analysis of target genes.

### 2.8. ELISA for Cytokine Secretion

For cytokine quantification, Ker-CT cells were seeded at 8.0 × 10⁴ cells/insert in 12-well transwell format, and supernatants were collected after 24 h treatment for ELISA analysis. TNF-α, IL-1β, and IL-18 levels in culture supernatants were measured by ELISA. Cytokine concentrations were calculated from standard curves and expressed as pg/mL or normalized percentage of control.

### 2.9. AGK2-Alone Control Groups

To determine whether AGK2 intrinsically modulates basal inflammatory or antioxidant signaling, AGK2-only groups (without DNCB stimulation) were included in parallel for major outcome measures including NF-κB pathway readouts, ROS quantification, and Nrf2 nuclear–cytoplasmic localization.

### 2.10. Protein Extraction and Western Blotting

Whole-cell lysates were prepared in RIPA buffer. Nuclear and cytoplasmic extracts were prepared where indicated. Proteins were resolved by SDS–PAGE and probed for SIRT2, Ac-α-tubulin, NF-κB p65, phospho-p65 (Ser536), IκBα, NLRP3, Nrf2, HO-1, Keap1, Laminin, GAPDH, and SOD1. Densitometry was performed using ImageJ (25).

### 2.11. Nuclear–Cytoplasmic Fractionation

Fractionation was performed using lysates from 3.0 × 10⁵ cells per condition (6-well format). Subcellular fractionation was performed to assess Nrf2 and p65 localization. Fraction purity was confirmed using Lamin B1 (nuclear) and GAPDH (cytoplasmic) markers.

### 2.12. Immunofluorescence and Confocal Microscopy

Ker-CT cells were seeded at 5.0 × 10⁴ cells/well on sterile glass coverslips in 24-well plates prior to treatment and staining. Ker-CT cells were fixed, permeabilized, and stained for NF-κB p65. Nuclei were counterstained with DAPI. Images were acquired under identical settings, and nuclear/cytoplasmic fluorescence ratios were quantified using ImageJ.

### 2.13. Statistical Analysis

Data are presented as mean ± SD from ≥ 3 independent experiments. Statistical analysis was performed using one-way ANOVA with Tukey’s post hoc test or unpaired Student’s t-test. A P-value < 0.05 was considered statistically significant.

## 3. Results

### 3.1. Establishment of an ACD-Like Inflammatory and Oxidative Stress Model in Ker-CT Keratinocytes

An in vitro allergic contact dermatitis–like model was established by exposing Ker-CT keratinocytes, cultured either alone or in transwell co-culture with CCD-1064Sk fibroblasts, to the prototypic sensitizer 2,4-dinitrochlorobenzene (DNCB). Nickel sulfate was included as a comparator allergen to validate the responsiveness of the system. This experimental setup was designed to recapitulate epidermal–dermal interactions relevant to allergic skin inflammation (Figure 1A).

DNCB exposure induced a significant, dose-dependent reduction in Ker-CT cell viability (Figure 1B). Compared with vehicle-treated controls (100%), low-dose (10 μM) and intermediate (25 μM) dose DNCB caused a modest but statistically significant decrease in viability that remained within the sub-toxic range (approximately 80 - 90%; P < 0.05 and P < 0.01). In contrast, high DNCB doses (50 μM) resulted in a pronounced loss of viability (P < 0.001), consistent with overt cytotoxicity at higher concentrations. Based on these findings, the intermediate DNCB concentration was selected for subsequent mechanistic experiments to induce inflammatory stress while preserving cellular integrity.

Analysis of the quantitative PCR data demonstrated strong activation of the inflammation pathway after exposure to the allergen (Figure 1C). There was a significant increase in the mRNA of the cytokines TNF-α and IL-1β in DNCB-treated cells compared with control cells (P < 0.01 for each). Nickel sulfate also caused a significant increase in these cytokines compared with control cells (P < 0.05 for each), although these were each significantly less than in DNCB-treated cells (P < 0.01, DNCB vs. nickel). This experiment validates the DNCB model as a strong sensitizer in the model described and the nickel sulfate model as an appropriate positive control, reflecting an ACD-like cytokine pattern. Given the stronger and more consistent magnitude of response elicited by DNCB in this system, DNCB was selected as the primary sensitizer for subsequent mechanistic analyses, whereas nickel sulfate was retained only as an early comparator to confirm model responsiveness.

Measurement of intracellular reactive oxygen species (ROS) by fluorescence intensity in Figure 1D revealed that Ker-CT keratinocytes exposed to DNCB had increased ROS as compared to control groups (P < 0.01). Importantly, Figure 1D directly compares DNCB-treated keratinocytes under monoculture versus transwell co-culture conditions, and ROS levels were significantly higher in the DNCB + fibroblast co-culture group than in the DNCB monoculture group (P < 0.05; DNCB co-culture vs. DNCB monoculture). The results indicate the importance of dermal and epidermal interactions in response to allergens in the generation of ROS. Accordingly, the increased oxidative burden observed in the co-culture condition provides experimental support that epidermal–dermal crosstalk amplifies hapten-induced redox stress in this model. Taken together, the results show that the proposed model has the ability to produce ACD conditions in the skin as well as to exhibit significant SIRT2 and Nrf2 expression. Collectively, these data establish a stable ACD-like model characterized by sub-toxic cellular stress, significant inflammatory cytokine induction, and enhanced oxidative burden, providing a robust platform for downstream investigation of SIRT2- and Nrf2-associated regulatory mechanisms.

### 3.2. SIRT2 Involvement in DNCB-Induced Inflammatory and Oxidative Stress Responses

**Figure 2.**
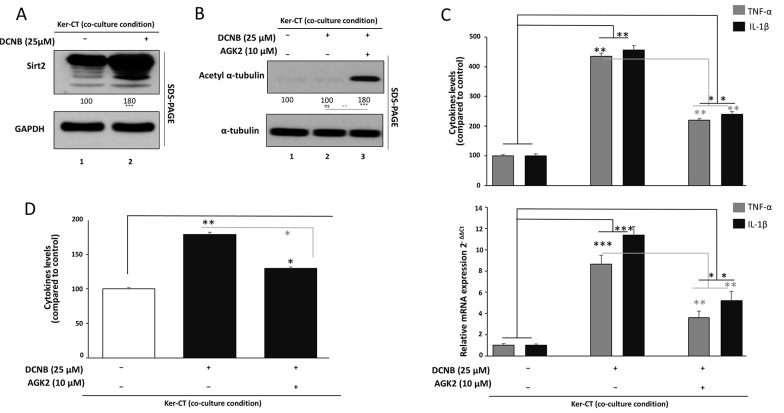
Involvement of SIRT2 in DNCB-induced inflammatory and oxidative stress responses A, SIRT2 protein levels in Ker-CT keratinocytes in transwell co-culture treated with DNCB (24 h) were assessed by Western blotting; GAPDH served as the loading control and densitometry was normalized to vehicle control. B, Functional target engagement was evaluated by acetylated α-tubulin (Ac-α-tubulin) accumulation after DNCB ± AGK2 treatment; total α-tubulin was used for normalization. C, Inflammatory responses were assessed by TNF-α and IL-1β mRNA levels (RT-qPCR; normalized to GAPDH) and secreted protein levels (ELISA) following DNCB ± AGK2. D, ROS generation was measured by DCFDA fluorescence following DNCB ± AGK2 and normalized to vehicle control. Data are mean ± SD from ≥ 3 independent biological experiments (n ≥ 3). Statistical testing: one-way ANOVA with Tukey’s post hoc test. Significance: *P < 0.05, **P < 0.01, ***P < 0.001. Where shown, horizontal comparison bars denote statistical contrasts (black bars: vs vehicle control; grey bars: vs DNCB alone).

Western blot analysis (Figure 2A) for Ker-CT keratinocytes cultured in transwells together with CCD-1064Sk fibroblasts revealed that DNCB induction increased SIRT2 protein expression in a repeatable manner. Compared with GAPDH normalization, SIRT2 protein expression was found to be upregulated by 1.5- and 1.8-fold in DNCB-treated cells, respectively (P < 0.001).

The sensitivity of SIRT2 protein expression, as detected by Western blot analysis in our experiments, confirms its responsiveness to the inflammation-like conditions created by DNCB exposure

In order to estimate functional SIRT2 activity, the cellular levels of Ac-α-tubulin were determined (Ac-α-tubulin) as a typical SIRT2 substrate (Figure 2B). Treatment with DNCB alone was associated with a minor reduction or preservation of basal Ac-α-tubulin levels relative to the control group (not significant, ns) to account for native SIRT2 deacetylase function. On the other hand, co-exposure with the specific SIRT2 inhibitor AGK2 caused a strong Ac-α-tubulin accumulation to reach around 1.5 to 1.8 times the control (P < 0.001 compared to DNCB alone). α-Tubulin was unaffected in all treatment groups (ns) to ascribe the detected differences solely to the differential acetylation status and not to variations in protein content. These findings validate the effectiveness of SIRT2 inhibition and its modulation by AGK2 in the DNCB-induced model of keratinocyte–fibroblast interaction.

In agreement with the inflammatory profile presented in Figure 1, DNCB exposure significantly enhanced TNF-α and IL-1β expression in co-cultured Ker-CT cells (Figure 2C). qPCR revealed several-fold upregulation of both cytokine transcripts compared to control (P < 0.01), with corresponding increases in secreted TNF-α and IL-1β protein levels to approximately 3 - 4-fold above baseline, as measured by ELISA (P < 0.001). Notably, AGK2 co-treatment significantly dampened the DNCB-induced cytokine expression, reducing both mRNA and protein levels to approximately 50 - 60% of the DNCB-alone group (P < 0.01 versus DNCB), yet remaining significantly higher than unstimulated controls (P < 0.05). This partial suppression suggests that SIRT2 inhibition modulates-but does not completely abrogate-DNCB-driven inflammatory signaling.

Analysis of intracellular ROS by DCFDA fluorescence (Figure 2D) revealed that DNCB strongly enhanced ROS generation in co-cultured keratinocytes to about 1.8-fold of control (P < 0.01). Co-treatment with AGK2 significantly diminished the DNCB-induced accumulation of ROS, decreasing levels to about 1.3-fold of control (P < 0.05 versus DNCB alone), but ROS levels remained higher than in untreated cells (P < 0.05). These results indicate that pharmacological inhibition of SIRT2 partly reestablishes the redox balance under ACD-like conditions.

### 3.3. SIRT2 Inhibition Attenuates DNCB-Induced NF-κB Activation in Ker-CT Keratinocytes

**Figure 3.**
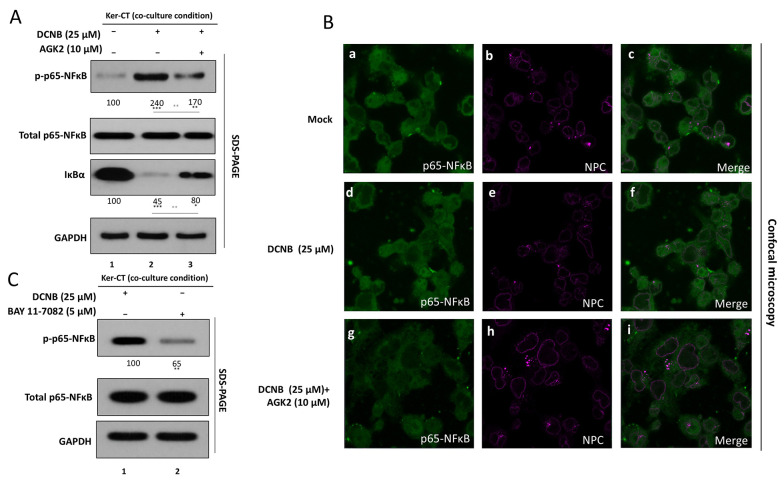
SIRT2 inhibition attenuates DNCB-induced NF-κB activation in Ker-CT keratinocytes A, NF-κB activation was assessed by Western blotting for phospho-p65 (Ser536) and total p65, presented as p-p65/p65 ratio, and for IκBα abundance following DNCB ± AGK2 treatment. Densitometric quantification was performed from ≥ 3 independent experiments and normalized to vehicle control. B, NF-κB p65 subcellular localization was assessed by immunofluorescence/confocal microscopy; representative images are shown and nuclear/cytoplasmic fluorescence ratios were quantified. C, Pharmacological validation using BAY 11 - 7082: p-p65/p65 ratios were compared across DNCB-treated groups with AGK2 or BAY 11 - 7082 by immunoblot and densitometry. Data are mean ± SD from ≥ 3 independent biological experiments (n ≥ 3). Statistical testing: one-way ANOVA with Tukey’s post hoc test. Significance: **P < 0.01. Where shown, horizontal comparison bars denote statistical contrasts (black bars: vs vehicle control; grey bars: vs DNCB alone).

As the DNCB-caused allergic contact dermatitis-like reaction in keratinocytes was associated with high cytokine secretion and oxidative stress (Figure 1), and partly alleviated by the pharmacological inhibition of SIRT2 activity (Figure 2), we chose to investigate if the NF-κB pathway, the central pathway of inflammation, is itself modulated by SIRT2 through its action on the NF-κB/IκB pathways (Figure 3A). In co-cultured Ker-CT cells using the transwell system, DNCB stimulation caused profound NF-κB activation as indicated by increased p-p65/total p65 levels in immunoblot analysis.Importantly, this effect was quantified by densitometric analysis (Figure 3A, quantification), with the p-p65/total p65 ratio increasing from 100% in untreated controls to 240% following DNCB exposure (P < 0.001). Inhibition of SIRT2 by the selective inhibitor AGK2 significantly reduced the densitometry-derived p-p65/total p65 ratio to 170% compared with DNCB alone (P < 0.01), indicating partial suppression of NF-κB activation. Total p65 protein abundance remained unchanged across groups (ns), as shown by immunoblot analysis and corresponding densitometric assessment. As NF-κB activates transcription as an ‘activation factor’ by binding IκB and its subsequent degradation via the ubiquitin-proteasome pathway, its induction by DNCB was associated with strongly diminished IκB. AGK2 co-treatment significantly restored IκBα expression toward baseline (P < 0.01 versus DNCB alone), although levels did not fully return to those observed in untreated cells (still P < 0.05 versus control). This partial recovery parallels the attenuation of p-p65 observed and supports stabilization of IκBα as a mechanism by which SIRT2 inhibition restrains NF-κB signaling.

Immunofluorescence analysis revealed predominantly cytoplasmic localization of p65 in control keratinocytes (Figure 3B, top panel). DNCB exposure induced prominent nuclear accumulation of p65 (Figure 3B, middle panel). AGK2 co-treatment significantly reduced nuclear p65 localization and partially restored cytoplasmic distribution (Figure 3B, lower panel).

Next, to verify NF-κB as a critical downstream effector of SIRT2 in this model, Ker-CT cells were treated with DNCB in the presence of a pharmacological NF-κB inhibitor BAY 11 - 7082 (5 μM) (Figure 3C). DNCB alone produced a high p-p65/p65 ratio, whereas co-treatment with the NF-κB inhibitor significantly suppressed p65 phosphorylation (P < 0.01 versus DNCB). Notably, the magnitude of reduction was comparable to that observed with AGK2 treatment in Figure 3A, indicating that direct NF-κB blockade phenocopies the effects of SIRT2 inhibition. These findings support the conclusion that attenuation of NF-κB activation and nuclear signaling is a major mechanism underlying the anti-inflammatory effects of SIRT2 inhibition in DNCB-stimulated keratinocytes.

### 3.4. SIRT2 Inhibition Suppresses NLRP3 Inflammasome Priming–Associated Responses and Restores Nrf2-Dependent Antioxidant Signaling in DNCB-Stimulated Keratinocytes

**Figure 4.**
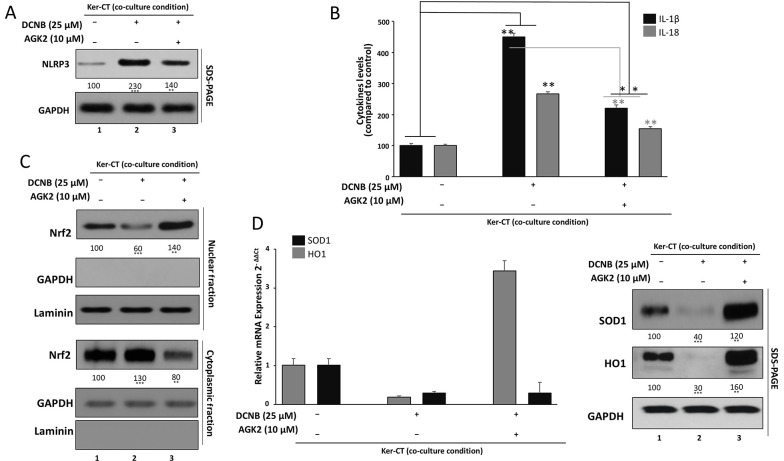
SIRT2 inhibition suppresses NLRP3 inflammasome priming–associated responses and restores Nrf2-dependent antioxidant signaling A, NLRP3 expression was assessed by Western blotting in Ker-CT keratinocytes treated with DNCB ± AGK2; densitometry was normalized to vehicle control. B, IL-1β and IL-18 secretion in culture supernatants was quantified by ELISA following DNCB ± AGK2. C, Nrf2 localization was assessed by nuclear–cytoplasmic fractionation and Western blotting; nuclear and cytoplasmic Nrf2 were normalized to Lamin B1 and GAPDH, respectively, and expressed as nuclear-to-cytoplasmic (N/C) ratio. D, Antioxidant outputs were assessed by HO-1 and SOD expression at mRNA level (RT-qPCR; normalized to GAPDH) and protein level (Western blot; densitometry normalized to vehicle control). Data are mean ± SD from ≥ 3 independent biological experiments (n ≥ 3). Statistical testing: one-way ANOVA with Tukey’s post hoc test. Significance: *P < 0.05, **P < 0.01, ***P < 0.001. Where shown, horizontal comparison bars denote statistical contrasts (black bars: vs vehicle control; grey bars: vs DNCB alone).

In Ker-CT keratinocytes maintained in transwell co-culture with CCD-1064Sk fibroblasts, DNCB exposure led to a clear induction of NLRP3 inflammasome components (Figure 4A). Western blot analysis showed low basal NLRP3 expression in control cells, set as 100% for normalization. Upon DNCB treatment, NLRP3 band intensity increased to approximately 2.3 - 2.8-fold of control across independent experiments, indicating robust inflammasome priming under ACD-like conditions. Co-treatment with the SIRT2 inhibitor AGK2 significantly lowered NLRP3 expression compared with DNCB alone, reducing normalized levels to roughly 1.4 - 1.8-fold of control rather than fully returning to baseline. This partial but consistent decrease suggests that SIRT2 activity contributes to sustaining NLRP3 upregulation in DNCB-challenged keratinocytes and that AGK2 dampens NLRP3 priming–associated inflammatory signaling rather than completely switching it off.

To determine whether increased NLRP3 expression was accompanied by enhanced inflammasome-associated IL-1 family cytokine output, we quantified IL-1β and IL-18 secretion by ELISA in culture supernatants from the same co-culture system (Figure 4B). In control cells, IL-1β and IL-18 were detected at low basal levels (∼20 - 25 pg/mL and ∼39 - 42 pg/mL, respectively, normalized to 100%). DNCB treatment caused a strong increase in IL-1β secretion, reaching mean values close to 4.5-fold of control, consistent with robust inflammasome-associated IL-1β output under DNCB challenge. IL-18 showed a similar trend, rising to roughly 2.6 - 2.8-fold of control following DNCB exposure. Importantly, AGK2 co-treatment significantly reduced both IL-1β and IL-18 release compared with DNCB alone, decreasing IL-1β to around 2.1 - 2.3-fold and IL-18 to approximately 1.5-fold of basal levels. Although cytokine levels in the AGK2 group remained higher than in untreated controls, the marked reduction relative to DNCB indicates that SIRT2 inhibition attenuates NLRP3 priming–associated IL-1β/IL-18 responses. Together with Panel 4A, these data support a model in which SIRT2 promotes DNCB-induced NLRP3 inflammasome priming and inflammasome-associated IL-1β/IL-18 release.

We next assessed whether SIRT2 affects Nrf2-driven antioxidant signaling using nuclear–cytoplasmic fractionation followed by Western blotting (Figure 4C). In vehicle-treated keratinocytes, Nrf2 displayed a balanced distribution between cytoplasmic and nuclear fractions, resulting in a nuclear-to-cytoplasmic (N/C) Nrf2 ratio close to 1.0 when normalized to GAPDH (cytosolic marker) and Lamin B1 (nuclear markers). DNCB exposure disrupted this pattern: nuclear Nrf2 levels dropped to approximately 60 - 70% of control, while cytoplasmic Nrf2 increased to about 130 - 150% of control, leading to a pronounced decrease in the N/C Nrf2 ratio to roughly 0.4 - 0.5. These changes indicate that, despite generating oxidative stress, DNCB impairs effective Nrf2 nuclear accumulation and may blunt the endogenous antioxidant response. In contrast, co-treatment with AGK2 reversed this imbalance. Nuclear Nrf2 was restored and modestly enhanced, rising to approximately 120 - 140% of control, whereas cytoplasmic Nrf2 decreased toward or slightly below baseline (∼80 - 90% of control). Consequently, the N/C Nrf2 ratio increased to about 1.3 - 1.4, exceeding that of unstimulated cells. This shift toward nuclear enrichment suggests that SIRT2 inhibition reactivates Nrf2 signaling in DNCB-challenged keratinocytes, providing a mechanistic link between SIRT2 blockade and improved redox regulation.

To confirm that Nrf2 reactivation translates into enhanced antioxidant capacity, we examined the expression of the Nrf2-responsive enzymes heme oxygenase-1 (HO-1) and superoxide dismutase (SOD) (Figure 4D). At the transcript level, qPCR analysis showed that DNCB reduced HO-1 and SOD1 mRNA to approximately 40 - 60% of control, in line with impaired Nrf2 nuclear signaling. In contrast, AGK2 co-treatment significantly upregulated both genes compared with DNCB alone, elevating HO-1 and SOD1 expression to values that approximated or slightly exceeded control (often in the range of 1.2 - 1.5-fold of baseline). Western blot analysis mirrored these transcriptional changes: DNCB exposure was associated with a marked decline in HO-1 and SOD protein levels when normalized to β-actin or GAPDH, consistent with weakened antioxidant defense in the ACD-like setting. Cells treated with DNCB plus AGK2 showed a clear restoration of HO-1 and SOD protein expression, with band intensities rising well above the DNCB-alone group and approaching or surpassing those of untreated controls. When plotted together, HO-1 and SOD followed a similar pattern of DNCB-induced downregulation and AGK2-mediated rescue. These findings indicate that SIRT2 inhibition does not merely rebalance Nrf2 localization (Panel 4C) but functionally restores downstream antioxidant enzymes, thereby supporting a more robust defense against DNCB-triggered oxidative stress.

### 3.5. Genetic Validation of SIRT2 and Assessment of AGK2-Alone Baseline Effects (A–D)

**Figure 5.**
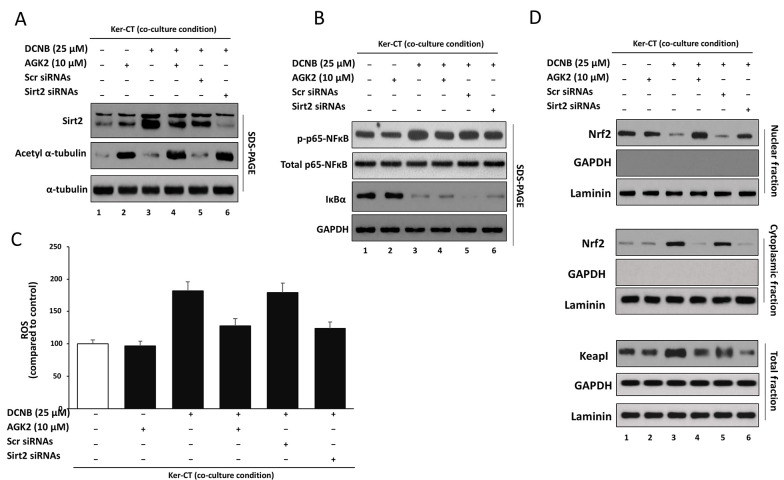
Genetic validation of SIRT2 involvement and assessment of AGK2-alone baseline effects in DNCB-stimulated Ker-CT keratinocytes Ker-CT keratinocytes were maintained in transwell co-culture with CCD-1064Sk fibroblasts and assigned to: G1 vehicle control; G2 AGK2 alone; G3 DNCB; G4 DNCB + AGK2; G5 DNCB + siCtrl; G6 DNCB + siSIRT2. A, SIRT2 knockdown and target engagement were verified by Western blotting for SIRT2 and Ac-α-tubulin; total α-tubulin and GAPDH served as loading controls; densitometry was normalized to vehicle control. B, Basal and stimulated NF-κB signaling was assessed by Western blotting for phospho-p65 (Ser536), total p65, and IκBα. C, ROS levels were quantified by DCFDA fluorescence and normalized to vehicle control. D, Nrf2–Keap1 axis: Nrf2 localization was assessed by nuclear–cytoplasmic fractionation, and Keap1 abundance was assessed in whole-cell lysates. Data are mean ± SD from ≥ 3 independent biological experiments (n ≥ 3). Statistical testing: one-way ANOVA with Tukey’s post hoc test. Where shown, horizontal comparison bars denote statistical contrasts (black bars: vs vehicle control; grey bars: vs DNCB alone).

To strengthen target specificity beyond pharmacological inhibition and to determine whether AGK2 intrinsically alters key pathways, SIRT2 was silenced in Ker-CT keratinocytes (siSIRT2) and compared with siCtrl under DNCB challenge, while AGK2-only groups (without DNCB) were analyzed in parallel in the Ker-CT/CCD-1064Sk transwell co-culture system (Figure 5A–D).

Immunoblotting (Figure 5A) confirmed efficient SIRT2 depletion in siSIRT2-transfected cells compared with siCtrl, together with functional target engagement indicated by increased acetyl-α-tubulin. DNCB exposure elevated SIRT2 abundance relative to vehicle controls, whereas AGK2 did not measurably change total SIRT2 protein but increased acetyl-α-tubulin, consistent with inhibition of SIRT2 deacetylase activity. siSIRT2 produced a similar increase in acetyl-α-tubulin under DNCB stimulation, phenocopying the pharmacological effect, while total α-tubulin remained unchanged.

To assess baseline NF-κB regulation by AGK2, p65 phosphorylation and IκBα abundance were examined with and without DNCB (Figure 5B). AGK2 alone did not significantly alter basal p-p65/total p65 ratio or IκBα levels compared with vehicle, indicating that AGK2 does not intrinsically activate or suppress NF-κB signaling under unstimulated conditions. In contrast, DNCB robustly increased p65 phosphorylation and reduced IκBα, confirming NF-κB activation. Co-treatment with AGK2 significantly reduced DNCB-induced p65 phosphorylation and partially restored IκBα. Consistently, siSIRT2 reduced NF-κB activation compared with siCtrl under DNCB challenge, supporting a SIRT2-dependent contribution to DNCB-induced NF-κB signaling.

DCFDA-based ROS measurements (Figure 5C) showed that AGK2 alone did not significantly change basal ROS levels compared with vehicle control. DNCB markedly increased intracellular ROS, whereas AGK2 significantly attenuated ROS accumulation. Genetic depletion of SIRT2 similarly reduced DNCB-induced ROS compared with siCtrl, although ROS levels remained modestly elevated relative to baseline, indicating partial restoration of redox balance.

Nuclear–cytoplasmic fractionation (Figure 5D) demonstrated that AGK2 alone did not substantially alter Nrf2 localization at baseline. In contrast, DNCB reduced nuclear Nrf2 with concomitant cytosolic retention, resulting in a pronounced decrease in the nuclear-to-cytoplasmic Nrf2 ratio despite increased ROS, consistent with impaired antioxidant adaptation under ACD-like stress. Both AGK2 and siSIRT2 restored nuclear Nrf2 enrichment and increased the nuclear-to-cytoplasmic ratio relative to DNCB-alone conditions. In parallel, DNCB increased Keap1 protein levels, which were partially normalized by AGK2 and siSIRT2, providing mechanistic support for dysregulated Keap1–Nrf2 signaling contributing to the ROS–Nrf2 paradox.

Together, these results provide genetic validation of SIRT2 involvement and demonstrate that AGK2 does not intrinsically modulate basal NF-κB, ROS, or Nrf2 signaling, supporting the conclusion that the observed protective effects of AGK2 reflect bona fide inhibition of SIRT2-dependent stress signaling in DNCB-stimulated keratinocytes.

### 3.6. SIRT2-Dependent Regulation of Inflammatory and Oxidative Stress Signaling in DNCB-Stimulated Ker-CT Keratinocytes

**Figure 6.**
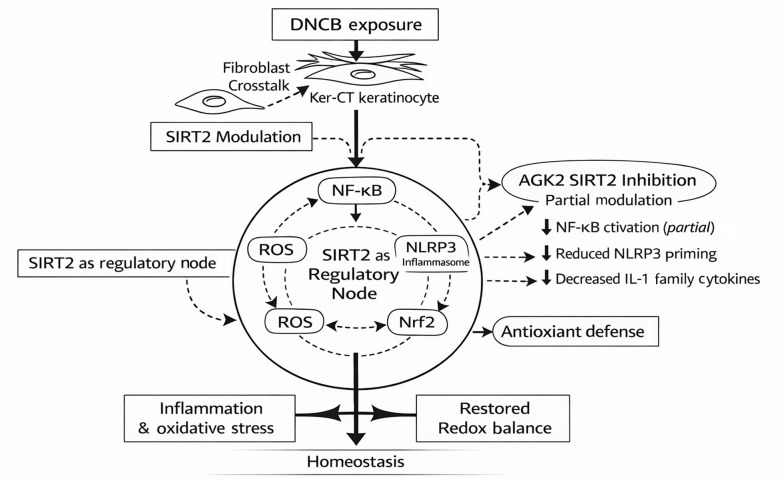
Flowchart summarizing SIRT2-dependent modulation of inflammatory and oxidative stress pathways in DNCB-stimulated keratinocytes Figure illustrating the sequence of molecular events identified in this study. DNCB exposure is associated with increased SIRT2 expression/activity in Ker-CT keratinocytes (enhanced by fibroblast–keratinocyte crosstalk), supporting NF-κB activation, NLRP3 priming–associated inflammatory outputs (including IL-1 family cytokine release), and increased oxidative stress, while reducing Nrf2 nuclear enrichment and downstream antioxidant responses. Pharmacological inhibition of SIRT2 by AGK2 partially attenuates NF-κB activation and dampens NLRP3 priming–associated IL-1 family cytokine responses, while promoting Nrf2 nuclear enrichment and antioxidant enzyme expression, resulting in partial rebalancing of inflammatory–redox signaling rather than complete normalization.

The schematic model (Figure 6) summarizes the integrated findings from Figure 1 - 5 and depicts SIRT2 as a regulatory node that modulates—rather than linearly dictates—hapten-induced inflammatory and redox pathways in Ker-CT keratinocytes. Following DNCB exposure, fibroblast–keratinocyte crosstalk amplifies the stress response, coinciding with increased SIRT2 expression/activity and activation of canonical inflammatory signaling. In this context, NF-κB signaling is enhanced (IκBα degradation, increased p65 phosphorylation, and nuclear p65 accumulation), driving induction of pro-inflammatory cytokines (e.g., TNF-α, IL-1β). NF-κB-associated signaling contributes to NLRP3 inflammasome priming–associated outputs, reflected by increased NLRP3 expression and elevated IL-1β/IL-18 secretion, alongside increased intracellular ROS. At the same time, DNCB is associated with impaired antioxidant adaptation, characterized by reduced Nrf2 nuclear enrichment and diminished expression of downstream antioxidant enzymes (HO-1, SOD), thereby reinforcing an inflammatory–oxidative stress state.

In contrast, AGK2-mediated SIRT2 inhibition is depicted as partial modulation of multiple interconnected nodes. Consistent with the experimental data, AGK2 partially attenuates NF-κB activation (reduced p65 phosphorylation and nuclear localization with stabilization of IκBα) and dampens NLRP3 priming–associated IL-1 family cytokine responses (reduced IL-1β/IL-18 output) rather than completely normalizing them. In parallel, SIRT2 inhibition supports restoration of Nrf2 nuclear enrichment with increased antioxidant gene/protein expression (HO-1, SOD), contributing to partial normalization of redox balance. Overall, Figure 6 is intended to convey regulatory complexity and stimulus-dependent, incomplete normalization (modulatory effects), aligning the schematic with the magnitude and directionality observed experimentally.

## 4. Discussion

This study identifies SIRT2 as a regulatory node linking inflammatory and redox signaling in a DNCB-driven, allergic contact dermatitis (ACD)–like human keratinocyte–fibroblast co-culture model. Using a physiologically relevant transwell system, we demonstrate that pharmacological inhibition of SIRT2 with AGK2 attenuates NF-κB activation and NLRP3 priming–associated IL-1 family cytokine output (IL-1β/IL-18), while simultaneously restoring Nrf2-dependent antioxidant defenses. These findings place SIRT2 at the intersection of hapten-induced stress, inflammation, and impaired cytoprotection in keratinocytes.

Consistent with established ACD pathogenesis, DNCB exposure induced pronounced oxidative stress and inflammatory signaling, evidenced by increased ROS generation, upregulation of TNF-α and IL-1β, and reduced keratinocyte viability at higher doses, thereby recapitulating early epidermal stress responses that precede adaptive immune activation (1 - 3). In parallel, nickel sulfate served as a comparator sensitizer in the validation arm and similarly increased TNF-α and IL-1β expression, confirming allergen responsiveness of the system (Figure 1C). Keratinocytes are increasingly recognized as active initiators of contact hypersensitivity through oxidative stress and cytokine release rather than passive barrier cells (9,26). Our co-culture findings further support the contribution of dermal–epidermal crosstalk in amplifying oxidative stress, a feature observed in both experimental and clinical dermatitis.

A central finding of this study is the robust induction of NLRP3 priming–associated inflammatory responses in DNCB-stimulated keratinocytes, reflected by increased NLRP3 expression and IL-1β/IL-18 secretion. NLRP3 inflammasome priming and inflammasome-associated IL-1 family signaling have emerged as key amplifiers of allergic and irritant skin inflammation, including contact hypersensitivity and atopic dermatitis, where IL-1 family cytokines shape downstream Th1/Th17-skewed responses and tissue injury (27 - 28). Pharmacological or genetic inhibition of NLRP3 has been shown to alleviate DNCB-induced dermatitis in vivo, underscoring its pathogenic relevance (14,29). Our human cell–based data extend these observations and provide mechanistic insight into how inflammasome priming is regulated upstream in keratinocytes.

Importantly, this work integrates SIRT2 into the NF-κB–NLRP3 inflammatory axis. SIRT2 is a cytosolic NAD⁺-dependent deacetylase known to regulate inflammatory signaling through modulation of NF-κB, cytoskeletal dynamics, and inflammasome components (19,20). In systemic inflammatory models, SIRT2 inhibition reduces cytokine release and improves outcomes in sepsis and infection-associated organ injury (30,31). Our observation that AGK2 reduces p65 phosphorylation, stabilizes IκBα, and partially suppresses inflammatory cytokine production in keratinocytes aligns with these reports and supports a pro-inflammatory role of SIRT2 in hapten-induced skin stress. Notably, the partial rather than complete normalization of inflammatory markers suggests that SIRT2 inhibition fine-tunes, rather than abolishes, innate immune signaling—potentially preserving essential host defense.

Another key contribution of this study is the demonstration that SIRT2 inhibition restores Nrf2-dependent antioxidant signaling under ACD-like conditions. Nrf2 is a master regulator of redox homeostasis in the skin, and its impairment exacerbates allergen-induced inflammation and NF-κB activation (18). We show that DNCB disrupts Nrf2 nuclear localization despite increased ROS, indicating functional suppression of antioxidant responses. This apparent paradox—elevated ROS concurrent with impaired Nrf2 nuclear accumulation—likely reflects dysfunctional redox adaptation rather than classical ROS-driven Nrf2 activation. Under conditions of sustained inflammatory or electrophilic stress, excessive ROS generation can coincide with impaired Nrf2 competence due to enhanced cytosolic sequestration, altered Keap1 regulation, or inflammatory crosstalk that interferes with Nrf2 stabilization and nuclear translocation. In our model, DNCB increased Keap1 expression and reduced the nuclear-to-cytoplasmic Nrf2 ratio, supporting a state of oxidative stress coupled with impaired antioxidant translocation rather than effective Nrf2 activation. Importantly, both pharmacological inhibition and genetic depletion of SIRT2 restored nuclear Nrf2 enrichment and partially normalized Keap1 levels, suggesting that SIRT2 contributes to dysregulated Keap1–Nrf2 signaling under inflammatory stress conditions. AGK2 reversed this defect, restoring nuclear Nrf2 and inducing HO-1 and SOD expression. These findings are consistent with reports in non-cutaneous tissues showing that sirtuin modulation can indirectly enhance Nrf2 activity and antioxidant capacity (32). Thus, SIRT2 inhibition appears to exert dual protective effects by dampening inflammatory signaling while reinforcing endogenous cytoprotection.

From a dermatologic perspective, this work adds to growing evidence that sirtuins contribute to epidermal inflammatory regulation and barrier homeostasis. While other sirtuins (e.g., SIRT1, SIRT3) have been implicated in psoriasis and skin aging (33,34), SIRT2 has not previously been mechanistically examined in a hapten-driven ACD model using human keratinocytes. By linking SIRT2 to NF-κB, NLRP3, and Nrf2 pathways in a human epithelial–stromal system, our study bridges systemic inflammatory models and skin-specific allergic disease.

Several limitations warrant consideration. Although AGK2 is widely used as a selective SIRT2 inhibitor, pharmacological inhibition alone may be influenced by off-target effects; therefore, we incorporated siRNA-mediated SIRT2 knockdown to strengthen causal inference and observed consistent phenocopying of the inhibitor effects across inflammatory, oxidative, and antioxidant readouts. However, we did not employ stable genetic manipulation (e.g., CRISPR-based SIRT2 knockout/knock-in) or rescue experiments (re-expression of SIRT2 following knockdown), which would further strengthen causal attribution of the observed effects specifically to SIRT2. In addition, AGK2-alone control groups were included to confirm that AGK2 does not intrinsically suppress basal NF-κB activity, ROS levels, or Nrf2 nuclear localization under unstimulated conditions, supporting that its major effects in this study reflect modulation of DNCB-induced stress signaling. Importantly, this work is limited to an in vitro keratinocyte–fibroblast co-culture system; thus, the pharmacological and mechanistic findings should be interpreted as model-based evidence, and in vivo validation in established DNCB- or nickel-induced dermatitis/contact hypersensitivity models is required to confirm physiological and therapeutic relevance. Although nickel sulfate was included as a comparator sensitizer for early model validation (Figure 1), downstream mechanistic analyses were intentionally focused on DNCB because it elicited a stronger and more reproducible inflammatory/oxidative phenotype in this system, providing a wider dynamic range for interrogating SIRT2-dependent NF-κB/NLRP3-associated and Nrf2 signaling; extension of these mechanistic analyses to nickel-driven responses will be valuable for assessing generalizability across sensitizer classes. Additionally, while IL-1β and IL-18 secretion together with increased NLRP3 expression are consistent with inflammasome priming–associated inflammatory responses, definitive confirmation of full inflammasome activation would require complex-level readouts such as cleaved caspase-1 and/or gasdermin D cleavage (and/or ASC speck formation).

In summary, our findings suggest that SIRT2 contributes to inflammatory–oxidative imbalance in hapten-challenged keratinocytes and that pharmacological inhibition with AGK2 is associated with restoration of a more balanced NF-κB–NLRP3 priming–associated IL-1 family cytokine response–Nrf2 signaling profile. Consistent with this, siRNA-mediated SIRT2 depletion partly phenocopied the effects of AGK2, supporting a role for SIRT2 in DNCB-driven inflammatory and redox dysregulation in this in vitro keratinocyte model. Collectively, these data indicate the potential therapeutic relevance of targeting SIRT2 as a host-directed strategy for allergic contact dermatitis–like inflammatory skin stress, warranting further validation in preclinical dermatitis models.

## Data Availability

The dataset presented in the study is available on request from the corresponding author during submission or after publication.
